# Mobile Phone Radiation Deflects Brain Energy Homeostasis and Prompts Human Food Ingestion

**DOI:** 10.3390/nu14020339

**Published:** 2022-01-14

**Authors:** Ewelina K. Wardzinski, Kamila Jauch-Chara, Sarah Haars, Uwe H. Melchert, Harald G. Scholand-Engler, Kerstin M. Oltmanns

**Affiliations:** Section of Psychoneurobiology, Center of Brain, Behavior and Metabolism, University of Luebeck, Ratzeburger Allee 160, 23538 Luebeck, Germany; kamila.jauch-chara@uksh.de (K.J.-C.); Sarah.Haars@medizin.uni-leipzig.de (S.H.); uwe.melchert@uni-luebeck.de (U.H.M.); kerstin.oltmanns@uni-luebeck.de (K.M.O.)

**Keywords:** mobile phone, radio frequency-modulated electromagnetic fields, brain, food intake, body weight

## Abstract

Obesity and mobile phone usage have simultaneously spread worldwide. Radio frequency-modulated electromagnetic fields (RF-EMFs) emitted by mobile phones are largely absorbed by the head of the user, influence cerebral glucose metabolism, and modulate neuronal excitability. Body weight adjustment, in turn, is one of the main brain functions as food intake behavior and appetite perception underlie hypothalamic regulation. Against this background, we questioned if mobile phone radiation and food intake may be related. In a single-blind, sham-controlled, randomized crossover comparison, 15 normal-weight young men (23.47 ± 0.68 years) were exposed to 25 min of RF-EMFs emitted by two different mobile phone types vs. sham radiation under fasting conditions. Spontaneous food intake was assessed by an ad libitum standard buffet test and cerebral energy homeostasis was monitored by ^31^phosphorus-magnetic resonance spectroscopy measurements. Exposure to both mobile phones strikingly increased overall caloric intake by 22–27% compared with the sham condition. Differential analyses of macronutrient ingestion revealed that higher calorie consumption was mainly due to enhanced carbohydrate intake. Measurements of the cerebral energy content, i.e., adenosine triphosphate and phosphocreatine ratios to inorganic phosphate, displayed an increase upon mobile phone radiation. Our results identify RF-EMFs as a potential contributing factor to overeating, which underlies the obesity epidemic. Beyond that, the observed RF-EMFs-induced alterations of the brain energy homeostasis may put our data into a broader context because a balanced brain energy homeostasis is of fundamental importance for all brain functions. Potential disturbances by electromagnetic fields may therefore exert some generalized neurobiological effects, which are not yet foreseeable.

## 1. Introduction

Mobile phone usage is an essential part of day-to-day communication in modern society. Thirty years ago, the first mobile phone started its triumphant advance around the world, leading to more than six billion subscriptions [1]. During the same time period, rising body weight and obesity in the human population spread worldwide [2] and according to the Global Burden of Disease study obesity is one of the leading risk factors for death globally [3]. In fact, one in five children and adolescents worldwide are overweight [4], and specifically mobile phone usage is strongly associated with overweight in children [5]. Although at first sight a simultaneous increase in mobile phone usage and body weight gain appears to be pure coincidence, the idea that they may be related is not as far-fetched as it seems [6]. Mobile phone-emitted radiation, i.e., radio frequency-modulated electromagnetic fields (RF-EMFs), is absorbed to more than 80% by the head [7], enters the human brain [8,9,10], increases circumscribed glucose turnover underneath the antenna [11], elevates cortical excitability upon motor-evoked potentials [12], and influences electroencephalogram (EEG) monitoring [13,14], as well as cognitive–motor processes [14] in humans. In children and adolescents, a recent comprehensive study by Birks and co-workers demonstrated that mobile phone calls on 2G networks are the main determinants of brain dose [15] and respective radiation is absorbed more in the child’s brain compared with adults [16,17]. Food intake regulation, in turn, is a function of hypothalamic and thereby central nervous appetite control [18]. Stimulation of the lateral hypothalamus drives feelings of appetite and initiates food intake while the ventromedial hypothalamus is responsible for satiety perception [19]. Daily RF-EMFs exposure for only 2 h—beginning in prepubertal rats—increase food intake and body weight three weeks later in the adult rodent [20]. Moreover, prolonged exposition to RF-EMFs over a time period of 5–22 weeks has been shown to continue body weight gain and foster food intake in rodents [21,22,23]. Against this background, we experimentally tested if acute exposure to mobile phone radiation influences food consumption in humans. Because pulsed, as well as amplitude-modulated microwaves have been shown to affect brain energy homeostasis in rats [24] and, on the other hand, the cerebral energy status itself seems to play a key role in food intake [25] and body weight regulation [26,27], we assumed that potential effects of mobile phone exposure on food consumption may be mediated through their influence on cerebral energy homeostasis.

## 2. Materials and Methods

### 2.1. Participants

We tested 15 healthy young men aged 21 to 29 years (mean ± s.e.m.: 23.47 ± 0.68 years) with a body mass index (BMI) of 20–25 kg/m^2^ (range: 21.5–24.5 kg/m^2^, mean BMI: 22.62 ± 0.32 kg/m^2^). All participants had a regular sleep-wake cycle for four weeks before testing. Exclusion criteria were acute or chronic internal, neurological, or psychiatric diseases, diabetes mellitus, obesity in 1st-degree family members, any kind of current medication, alcohol and/or drug abuse, smoking, shift work, and competitive sports. Before experimental testing, each volunteer gave written informed consent. Because sleep restriction enhances food consumption [28,29], participants were asked to go to bed no later than 11:00 p.m. before and during the experimental testing period. Additionally, participants were instructed to abstain from food and caffeine for 12 h before the experiments. No mobile phone use was allowed during the period of 12 h before the experiments, which was randomly controlled by test calls.

### 2.2. Study Design

**Figure 1:**
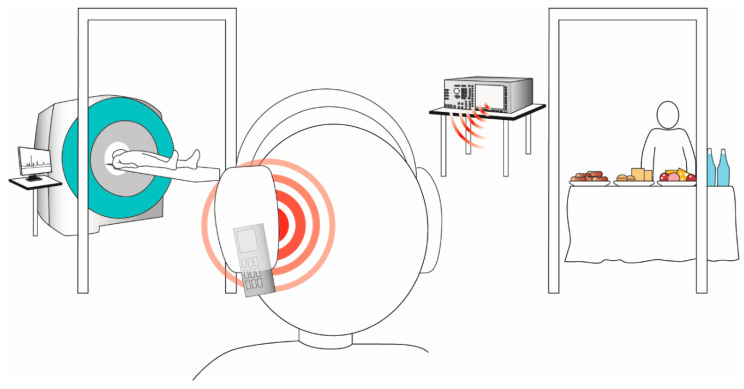
Schematic illustration of the laboratory setting. Individuals were exposed to two different continuously transmitting mobile phone types via a base station simulator working with maximal power vs. a deactivated phone as a sham condition. Mobile phones were installed in a compact headset without being visible to the participants to ensure that each subject exhibited the same distance between the mobile phone and the head. The antenna was located over the right temporal region. Participants were not given acoustic cues revealing the operation status of the mobile phones, i.e., they were unaware whether the phones were transmitting or not. Standardized buffet testing and MR-scanning procedure occurred in adjacent rooms.

The study was performed in a randomized, sham-controlled, single-blind, cross-over design. Each subject was tested on 3 experimental conditions for 25 min of exposure (mobile phone 1, mobile phone 2, and sham) spaced at least two weeks apart. On the days of experimental testing, participants reported to the Department of Neuroradiology at 6:30 a.m. after fasting for at least 12 h. To ensure that all participants were fasting at the start of the measurements, blood glucose levels were determined. No participants showed statistically significant differences in their fasting blood glucose levels (*p* = 0.322). One cannula was inserted into an antecubital vein for blood sampling of glucose, insulin, and C-peptide as known factors influencing glucose metabolism and appetite regulation. Thereafter, a baseline ^31^phosporus magnetic resonance spectroscopy (^31^P-MRS) sequence was recorded. Subsequently, participants were exposed to 5 min of sham/mobile phone radiation as described below. Immediately after this exposure, one ^31^P-MRS sequence was recorded to encompass potential direct effects of mobile phone exposure on brain energy metabolism. Thereafter, participants were exposed to additional 20 min of mobile phone or sham radiation. The duration of the exposure time was chosen regarding realistic and feasible aspects. Furthermore, studies are already available that provide guidance on appropriate stimulation times for anodal transcranial direct current stimulation in the context of high energy phosphates and food intake [30,31,32]. Subsequently, a series of 5 continuous ^31^P-MRS sequences was started. At 8:30 a.m., a standardized breakfast buffet [25,33] was offered from which participants were allowed to eat ad libitum during the subsequent 40 min. Participants were not informed about the actual purpose of the study to prevent them from putting a focus on food intake. Instead, they were told that the main intention of the study was to investigate the effects of mobile phone usage on cerebral energy metabolism. Thereby, participants were kept unaware of the hypothesized effects on food intake behavior and did not realize that their individual food consumption was quantified by weighing buffet components before and after the meal. To avoid overeating, participants were allowed to take with them any remaining food after final food weighing. Blood samples were obtained at baseline, at the end of the first mobile phone/sham phone exposure, at the end of the following spectroscopy recording, at the beginning and the end of the 20 min of mobile phone/sham phone exposure, at 10 min intervals during the 5 continuous spectroscopy sequences, and before and after the buffet test. Schematic illustration of the laboratory setting (Figure 1).

### 2.3. Mobile Phone Radiation Exposure

In order to increase the overall validity of our data, we used two arbitrarily chosen commercially available mobile phones (phone 1: Motorola L2 (Motorola Mobility LLC), Libertyville, IL, USA and phone 2: Nokia 5800d-1 (Nokia Group, Helsinki, Finland)) in our experiments. Both transmitted in the Global System for Mobile Communications (GSM) standard (900 MHz). Variances in RF-EMFs intensity due to environmental influence on phone reception quality were excluded by continuous transmission via a base station simulator (Universal Radio Communication Tester CMU 200, Rohde & Schwarz GmbH & Co. KG, Munich, Germany) working with maximal power [34,35]. Specific absorption rates (SAR) were 0.97 W/kg for phone 2 and 1.33 W/kg for phone 1. Participants reclined comfortably in a horizontal position on a stretcher. Mobile phones were installed in a compact headset without being visible to the participants. This setting ensured that at each subject exhibited the same distance between mobile phone and head. The antenna was located over the right temporal region. In the sham condition, a deactivated phone was installed. Participants were not given acoustic cues revealing the operation status of the mobile phones, i.e., they were unaware whether the phones were transmitting or not.

### 2.4. ^31^Phosphorus Magnetic Resonance Spectroscopy Measurements

^31^P-MRS measurements of the motor cortex were conducted in a 3.0 Tesla MR scanner (Achieva 3T, Philips Medical Systems, Best, Amsterdam, The Netherlands) using a double-tuned ^1^H/^31^P-headcoil (Advanced Imaging Research Inc., Cleveland, OH, USA). A repetition time of 4500 ms in conjunction with a three-dimensional chemical shift imaging (3D-CSI) sequence (6 × 5 × 3 voxel, 6 kHz bandwidth, 1024 data points, 8:51 min measuring time) was used to reach sufficient relaxation of the phosphorus metabolites. For a better spectral resolution during excitation, ^1^H-decoupling and nuclear Overhauser effect (NOE) were applied [36] with broadband proton decoupling (10 rectangular RF pulses at proton resonance frequency of 10 ms duration and 10 ms delay between each other to generate a 90° flip angle on the ^1^H nuclei). During receiving, ^1^H-decoupling (wideband alternating-phase technique for zero-residual splitting: WALTZ-4) [37] was applied for transmitting on the ^1^H-resonance frequency using the 2nd channel of the head coil. Magnetic Resonance User Interface (MRUI) was used for evaluation of the spectral data. Zerofilling to 4096 data points and apodizing with a 20 Hz-Lorentzian-filter were applied. Peak positions and intensities were calculated by AMARES-algorithm [38]. Adenosine triphosphate (ATP) was determined as the sum of alpha-, beta-, and gamma-ATP. The ratios of phosphocreatine (PCr)/inorganic phosphate (Pi) and ATP/Pi were evaluated as a common indicator of the intracellular energy status [31,39,40]. High-energy compounds are shown as single values at each time point. These values illustrate an arithmetic mean measured over all given voxels at a time point.

### 2.5. Statistical Analysis

Data are presented as mean values ± standard error of mean (s.e.m.). Statistical analysis using Superior Performing Software Systems (SPSS, IBM, Armonk, NY, USA) was based on analysis of variance for repeated measurements (ANOVA), including the factors ‘treatment’ (phone 1 vs. phone 2 vs. sham) and ‘time’ (time points of data collection), as well as the interaction effect between these factors. The suitability of ANOVA was determined by Mauchly’s sphericity test. In the case of violation of the sphericity conjecture, Greenhouse–Geisser correction was applied. For pairwise comparisons, paired Student’s *t*-test was used. A *p*-value less than 0.05 was considered significant.

## 3. Results

### 3.1. Food Ingestion

**Figure 2:**
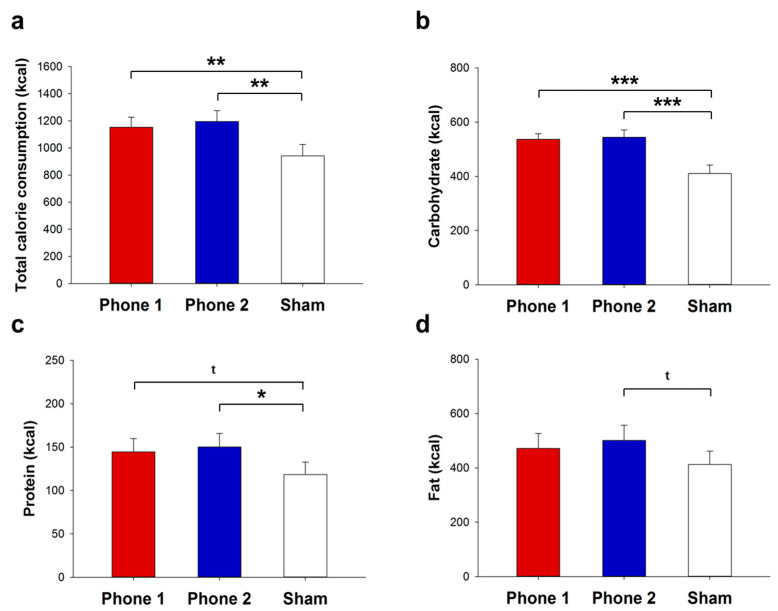
Calorie consumption after mobile phone exposure. Total calorie consumption (**a**), as well as ingested calorie content in the form of carbohydrates (**b**), proteins (**c**), and fat (**d**) from a standardized free-choice ad libitum buffet after 25 min of mobile phone (phone 1 (red bars) and phone 2 (blue bars) or sham (white bars)) exposure, respectively. Values are mean ± s.e.m.; two-tailed Student’s *t*-test; *n* = 15; * *p* < 0.05, ** *p* < 0.01, *** *p* < 0.001, ^t^ *p* < 0.1.

As hypothesized, we found that exposure to both transmitting mobile phones considerably increased total calorie consumption by 22% and 27% compared with the sham condition, respectively (phone 1: 1152.2 ± 75.1 vs. 941.6 ± 85.2 kcal, *p* = 0.001; phone 2: 1195.2 ± 79.3 vs. 941.6 ± 85.2 kcal, *p* = 0.001, Figure 2a). Strikingly, we found this clear radiation-induced food intake boost in thirteen participants, i.e., in nearly all of our participants. Comparisons between both phone types did not reveal any significant differences in terms of their impact on total calorie consumption (*p* = 0.326). Because appetite regulation is intrinsically tied to glucose metabolism, we monitored concentrations of blood glucose, serum insulin, and C-peptide throughout the study. Results show that all measures remained unaffected by mobile phone exposure (*p* > 0.117 for all analyses). Discrete analyses of the ingested macronutrients revealed that mobile phone radiation indeed enhanced carbohydrate (*p* < 0.001 for both phones, Figure 2b) and protein (phone 1: *p* = 0.067, phone 2: *p* = 0.014, Figure 2c) intake. Fat consumption increased as a trend after phone 2 exposure, while there was no such effect in the phone 1 condition (phone 1: *p* = 0.119, phone 2: *p* = 0.065, Figure 2d). Basically, there was no difference in terms of macronutrient content of the ingested food between both phones (*p* > 0.251 for all, Figure 2b–d). These findings indicate that the exposure to both active mobile phones not only boosted calorie intake in general but specifically made participants consume more calories in the form of carbohydrates and—to a lesser extent—proteins, i.e., participants favored a special composition of food after mobile phone use.

### 3.2. Cerebral High-Energy Phosphate Metabolism

**Figure 3:**
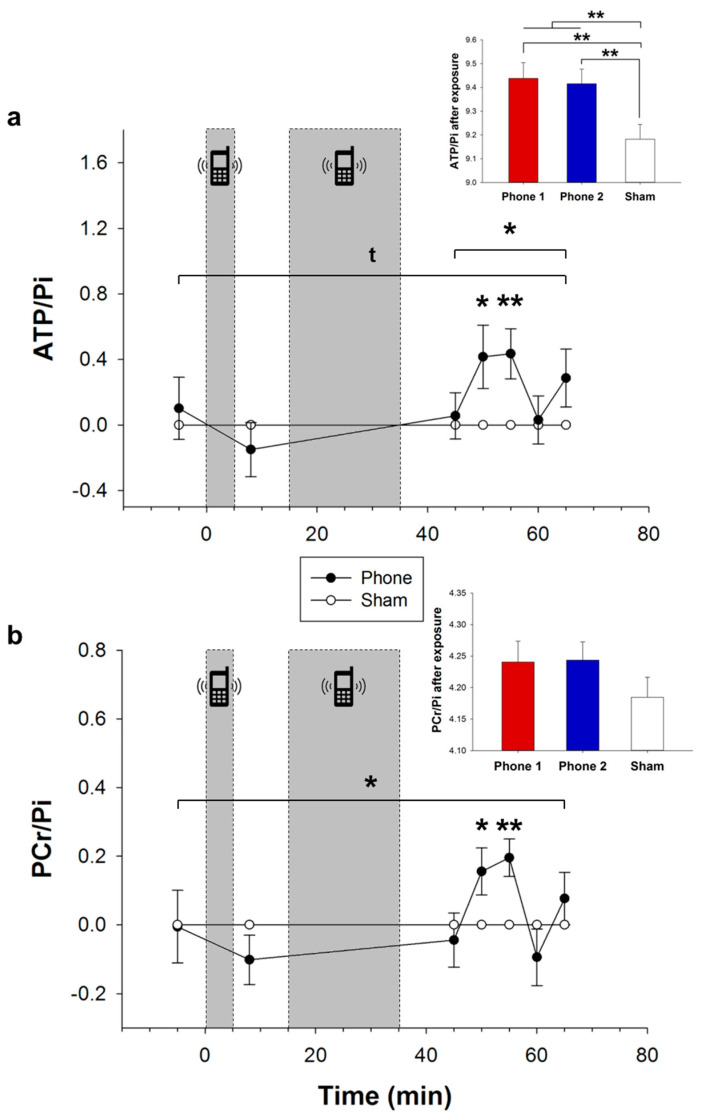
Effects of mobile phone exposure on neuroenergetic measures. Relative changes (mean values ± s.e.m.) of cerebral ATP/Pi (**a**) and PCr/Pi ratios (**b**) after 5 min and a subsequent further 20 min of mobile phone radiation (both phones merged, black dots) in proportion to respective sham condition values (white dots). Grey areas mark the intervention period of mobile phone or sham exposure. Bar charts compare mean values ± s.e.m. of phone 1 (red), phone 2 (blue), and sham (white) exposure, including all time points after intervention. * *p* < 0.05, ** *p* < 0.01, ^t^ *p* < 0.1.

Results of ATP/Pi and PCr/Pi ratios show comparable values for all experimental conditions at baseline (all *p* > 0.429), and after 5 min of mobile phone exposure (all *p* > 0.160). After the extended radiation procedure for a further 20 min, ATP/Pi levels were higher as a trend than after sham (*p* = 0.074, Figure 3a), an effect that was significant in terms of PCr/Pi ratios (*p* = 0.038, Figure 3b). Apparently, this was mainly due to differences at 50 and 55 min after radiation onset (*p* < 0.036 and *p* < 0.006, respectively, Figure 3a,b). Comparison of mean post-exposure ATP/Pi values revealed significantly higher levels after RF-EMFs radiation compared with sham (*p* = 0.009, Figure 3a, small insert). Differential analyses showed increased ATP/Pi levels by 3% after both phone 1 (*p* = 0.003) and phone 2 (*p* = 0.006) application than after sham, respectively (Figure 3a, small insert). These effects did not reach significance in terms of PCr/Pi ratios (*p* > 0.147, Figure 3b, small insert).

## 4. Discussion

Our data show that mobile phone radiation by two different phone types leads to higher calorie consumption by 22% and 27%, respectively. This effect was mainly based on an increased consumption of carbohydrates. This surprisingly unequivocal result is in line with scarce previous data from rodent studies, which examined food intake behavior upon RF-EMFs exposure. In fact, Tripathi and co-workers recently found an increased food intake after three weeks of a 2 h daily mobile phone exposure and consequent weight gain in rodents [20]. In another study, Lovely and co-workers observed an increased chow intake after RF-EMFs application of a comparable radiation intensity (915 MHz) to our own experiment [41]. Pelletier and co-workers confirmed this result and demonstrated that continuous RF-EMFs exposure for five weeks causes an increased daytime intake of standard chow by 0.22 g/h compared with a non-stimulated control group [23]. However, all data gained from rodents must be considered with caution when compared with our human experiments because RF-EMFs exposure generally irradiates the entire organism of small animals, leading to a considerable systemic heat supply which, in turn, increases the temperature by 0.5 °C [42] and thereby the basal metabolic rate of the whole organism. Such systemic effect certainly influences food intake behavior per se. In contrast, our experimental setting of applying genuine mobile phone radiation for a realistic duration of 25 min within a circumscribed activity area close to the brain corresponds to realistic conditions and does not exert such thermal influence. Moreover, physical activity during the experiments may also be considered as a bias in animal studies. However, there are also human enquiries, which suggest a close relationship between RF-EMFs exposure and body mass index (BMI), as explored using anthropometric measurements and self-reported questionnaires in children [5] and from more than 4000 Finnish adolescent twins [43]. Due to this evidence, the relationship between RF-EMFs exposure and increased food consumption in our study deserves closer inspection. In addition to the increased food intake after mobile phone radiation, analyses of the distribution of ingested macronutrients revealed that exposure to both active mobile phones, not only boosted calorie intake in general, but specifically made participants consume more calories in the form of carbohydrates and—to a lesser extent—proteins. This corresponds with our previous work, which experimentally revealed that both the intranasal application of insulin and transcranial direct current stimulation suppress human calorie consumption specifically due to a lowered ingestion of carbohydrates [25,44] and proteins [25]. In conclusion, it is suggested that human carbohydrate intake is unconsciously susceptible to central nervous appetite signaling. In line, hypothalamic ghrelin level is increased, while glycolysis (hexokinase) is lower upon exposure to mobile phone radiation in rats [20], indicating a disturbed appetite regulation. Furthermore, current findings from Kim and co-workers demonstrate alterations in the postsynaptic structure and neurite outgrowth constraints on the hippocampal neurons of early postnatal mice [45]. The hypothalamus and hippocampus are crucially involved in the regulation of food intake and body weight [45,46,47,48,49], and both are affected by RF-EMFs radiation already in young rodent brains [20,45]. Moreover, in 5- and 10-year-old children, RF-EMFs exposure penetrates the brain far deeper than the mid-brain [50], reaching the hypothalamus amongst others [17]. An influence of RF-EMFs exposure on neuroenergetic functioning is particularly interesting as the hypothalamic glucose-sensing neurons respond to changes in cytosolic ATP levels, and carbohydrates, mostly in the form of glucose, play a crucial role as substrates of cerebral energy generation, i.e., brain adenosine triphosphate (ATP) synthesis. In line with this assumption, we found in our neuroenergetic measurements that RF-EMFs exposure affects cerebral high-energy phosphate homeostasis. In former studies, we found that obese individuals generally display reduced cerebral levels of the high-energy phosphates ATP and PCr [39,40], the neuroenergetic status negatively correlates with food intake behavior [25], and the brain’s high-energy phosphate content even predicts the amount of subsequently consumed calories [25]. However, with regard to the previously observed inverse relationship between neuroenergetic status, food consumption [25], and BMI [39,40], one would have rather expected a drop than an increase in high-energy phosphate content. On the other hand, the apparent enhancement in ATP synthesis rate upon RF-EMFs may have required some kind of compensational glucose replenishment to sustain the neuroenergetic homeostasis thereafter. This would be in line with findings of increasing brain glucose metabolism after 50 min of mobile phone exposure in healthy humans [11], and elevated hypothalamic gluconeogenesis in rats [20]. Therefore, it can be speculated in our study, that the boosted carbohydrate consumption after mobile phone exposure may have served to satisfy this elevated neuroenergetic glucose need after radiation exposure. We previously observed a similar effect during a stress intervention and after hypocaloric dieting in humans. Under both conditions, high-energy phosphates and food intake increased [27,33]. In fact, 60 min of mobile phone RF-EMFs exposure modulates the NADPH oxidase and induces a significant increase in mitochondrial membrane potential in human astrocytes [51]. These findings indicate that the use of mobile phones has an impact on the mitochondrial respiratory chain, which in turn is essential for ATP synthesis and is in line with very early data in rats showing that mitochondria utilize energy from an applied electric field to synthesize ATP [52]. However, the significance of the observed RF-EMFs-induced influence on the central nervous system may not be limited to food intake behavior. Alterations in brain energy homeostasis as observed here may exert a number of additional effects on brain function such as neurocognitive performance, mental health maintenance, or behavioral control [53,54]. This is particularly true since the influence of RF-EMFs is not restricted to single regions of the brain [15] but exerts some generalized neurobiological effects. Previous findings showed an association between mobile phone use and headache/migraine, concentration difficulties, fatigue, skin itches, and sleep disturbances in children and high school students [55,56]. We performed our experiments with young healthy adults but of course our findings lead to far-reaching considerations about the effects of mobile phone use in children as well. Early usage during childhood or even owning a mobile phone at young age leads to radiation exposure for a significantly longer period in their lives than all generations had experienced before [57,58]. On the one hand, children’s brains absorb significantly higher radiation dosages emitted by mobile phones and RF-EMFs affect deeper brain structures (e.g., the hypothalamus) compared with adults [15,17,50]. On the other hand, childhood obesity is one of the most serious global public health challenges of the 21st century [59]. Against this background, our findings of increased food intake and deflected cerebral energy homeostasis upon mobile phone use give cause for concern, specifically in children and adolescents. However, it must be mentioned that some aspects of our study may limit the interpretation of our data. One may argue that, in our study, we tested rather antiquated types of mobile phones (which occurred on purpose to prevent harm to the producers’ businesses), but a respective SAR between 0.97 W/kg and 1.33 W/kg is absolutely comparable to modern mobile phone types [60], and was in accordance with the guidelines from the International Commission on non-ionizing radiation protection [61]. It is well known that sleep restriction increases daily fat, protein, and carbohydrate intake, as well as the daily energy intake, in humans [62]. Furthermore, hypothalamic ATP levels decrease during sleep deprivation and strongly correlate with food intake [63] We, therefore, excluded participants with shift work and irregular sleep patterns and questioned them prior to testing about sleep disturbances during the preceding night. However, because we did not monitor sleep under controlled laboratory conditions, we cannot exclude the possibility that individual participants slept poorly or not at all before the measurements and did not communicate this with us. In addition, emotional stress can also affect food intake [64]. However, it must be considered that all participants were tested on three different days at least two weeks apart, so it is questionable as to whether such a transient variable can explain our significant results. By all means, in the context of the current obesity epidemic, our findings of mobile phone-induced enhancement in consumed calories by more than 22% in young men seems alarming, particularly since the underlying reasons of why people overeat despite the awareness of harmful consequences are still unknown. Furthermore, it remains to be clarified to what extent head-distant activities such as messaging also have an impact on human food intake. Environmental influence of RF-EMFs as also derived from electronic devices, such as wireless LAN, Bluetooth, radio, and television adding to a dysfunction of hypothalamic appetite regulation would offer a conceivable explanation here.

## 5. Conclusions

Our human study demonstrates that the RF-EMFs radiation emitted by mobile phones results in significantly increased food ingestion, particularly carbohydrate intake. Moreover, a deflected cerebral high-energy phosphate metabolism, which is closely related to food intake and body weight, was found after mobile phone use. Therefore, our results identify RF-EMFs as a potential contributing factor to overeating in humans, which underlies the worldwide obesity epidemic. Beyond this, RF-EMF-induced alterations of the brain energy homeostasis, as observed here, may put our data into a broader context because a balanced central nervous energy homeostasis is of fundamental importance, not only for the regulation of food intake and body weight, but also for all brain functions. Therefore, with good cause, the high priority of this research field was already emphasized a long time ago by the WHO Research Agenda for Radiofrequency Fields [65]. Perhaps our data could serve as first step towards deeper insight into this issue and open a new perspective in neurobiological and obesity research.

## Data Availability

Data are contained within the article and could be presented on request.
